# Characteristics of initiation timing and anticoagulation of continuous renal replacement therapy in patients following cardiac surgery: A retrospective analysis of 28 patients

**DOI:** 10.1097/MD.0000000000039466

**Published:** 2024-09-06

**Authors:** Jian Li, Yi Li, Xianglian Li, Liwen Mo, Fan Zhang, Yue Cheng, Tao Wang

**Affiliations:** a Department of Nephrology, The General Hospital of Western Theater Command, Chengdu, Sichuan Province, China.

**Keywords:** cardiac surgery-associated acute kidney injury, continuous renal replacement therapy, low molecular weight heparin, regional citrate anticoagulation

## Abstract

Continuous renal replacement therapy (CRRT) used in cardiac surgery-associated acute kidney injury (CSA-AKI) may have different characteristics from other diseases. We reviewed the medical records of patients with CSA-AKI requiring CRRT who underwent cardiac surgery from January 2020 to September 2021. Patients with AKI caused by other reasons who received CRRT during the same period were also evaluated. A total of 28 patients with CSA-AKI and 12 patients with AKI caused by other reasons were enrolled in this study. Compared with AKI patients caused by other reasons, patients with CSA-AKI were found to have lower mean arterial pressure, higher level of bilirubin, higher vasoactive-inotropic score, and larger daily diuretic dosage. The patients with CSA-AKI were prescribed CRRT earlier than the patients with AKI caused by other reasons. There was a significant difference in the CRRT anticoagulation method between patients with CSA-AKI and patients with AKI caused by other reasons. Six patients with CSA-AKI were treated with regional citrate anticoagulation (RCA), and the other 22 patients were treated with low molecular weight heparin or without anticoagulants. The timing of CRRT initiation in patients with CSA-AKI is earlier than that in patients with AKI caused by other reasons. Although RCA is recommended as the preferred anticoagulant for patients without contraindications, patients with CSA-AKI often have circulatory dysfunction and severe liver damage, so the risk of citrate accumulation is greater, whether to use RCA should be determined according to the individual condition of the patient.

## 1. Introduction

The risk of acute kidney injury (AKI) after cardiac surgery has been reported to be as high as 20% to 50%, with 2% of these patients requiring renal replacement therapy.^[1–5]^ Continuous renal replacement therapy (CRRT) is recommended for patients with hemodynamic instability and fluid overload intolerance.^[6]^ Following cardiac surgery, patients are prone to low cardiac output, hemodynamic instability, a high risk of secondary infection, and the use of anticoagulants such as warfarin. Therefore, the characteristics of CRRT used in these patients may differ from those of patients with other diseases. The demographic characteristics, laboratory data, and CRRT prescriptions of patients who underwent CRRT for cardiac surgery-associated AKI (CSA-AKI) in our hospital between January 2020 and September 2021 were analyzed retrospectively. Differences in the clinical data between patients with AKI after cardiac surgery and those with AKI without cardiac surgery were analyzed. In addition, the side effects and effectiveness of different anticoagulation methods were compared to provide a reference for the implementation of CRRT in patients with CSA-AKI.

## 2. Patients and methods

### 2.1. Study subjects

We reviewed the medical records of patients with CSA-AKI requiring CRRT who underwent cardiac surgery between January 2020 and September 2021. Patients who had renal dysfunction before cardiac surgery, who refused when CRRT was recommended, patients on extracorporeal membrane oxygenation, or who died within 24 hours after cardiac surgery were excluded. Patients with AKI caused by other reasons who received CRRT during the same period were also evaluated.

The diagnostic criteria for AKI referred to the Kidney Disease Improving Global Outcomes consensus. This retrospective study was carried out using the opt-out method for the case series of our hospital. The study was approved by the local Ethics Committee and was conducted in accordance with the 1964 Helsinki Declaration and its later amendments or comparable ethical standards. Informed consent was waived by our Institutional Review Board because of the retrospective nature of our study.

### 2.2. Data collection

The baseline demographic and clinical characteristics (heart disease status, cardiopulmonary bypass time, intra-aortic balloon pump use, blood pressure, urine volume, sequential organ failure assessment score, laboratory indicators, and use of CRRT) of the patients were recorded. Blood samples were drawn before starting CRRT. The platelet count and serum levels of hemoglobin, creatinine, alanine aminotransferase, bilirubin, procalcitonin, and C-reactive protein were measured using automated and standardized methods at a centralized laboratory.

### 2.3. Statistical analysis

The measurement variables are presented as medians (P25, P75), and the count variables are described as frequencies and percentages. The Mann–Whitney *U* test was used to compare the medians of the measurement variables, and Fisher exact test was used to compare the count variables. All of the tests were 2-sided, and differences were considered significant at *P* < .05. All of the statistical analyses were performed using SPSS software, version 22.

## 3. Results

### 3.1. Demographic and clinical characteristics of patients with CSA-AKI who received CRRT

A total of 28 patients with CSA-AKI (17 with valvular heart disease, 5 with aortic dissection, 1 with coronary heart disease, 2 with infective endocarditis, and 3 with congenital heart disease) and 12 patients with AKI caused by other reasons (2 patients with acute pancreatitis, 1 patient with cytokine release syndrome, 2 patients with severe pneumonia, 2 patients with craniocerebral injury, 2 patients with crescent nephritis, 1 patient with wasp sting, and 2 patients with coronary heart disease) were enrolled in this study. All patients agreed to CRRT. The demographic and clinical characteristics as well as laboratory results are summarized in Table 1.

**Table 1:** Comparison of demographic and clinical characteristics between patients with CSA-AKI and Patients with AKI caused by other reasons.

Characteristics and measure	Cardiac surgery-associated AKI (N = 28)	AKI without cardiac surgery (N = 12)	Z	*P* value
Sex (n)	Male 14	Male 9		.179
Age (years)	56.00 (50.00, 63.00)	66.00 (60.00, 83.00)	−1.462	.148
BMI	22.43 (20.03, 25.51)	21.64 (19.53, 22.09)	−0.903	.377
Hemoglobin (g/L)	106.00 (100.00, 120.00)	72.00 (63.00, 89.00)	−3.691	.000
Platelets (×10^9^/L)	114.00 (79.00, 175.00)	139.00 (85.00, 169.00)	−0.458	.657
Creatinine (μmol/L)	205.00 (139.00, 278.00)	482.70 (340.00, 665.00)	−3.807	.000
PCT (ng/mL)	3.82 (1.69, 11.76)	2.61 (0.49, 9.21)	−1.033	.313
CRP (mg/L)	79.47 (48.87, 153.46)	79.14 (46.09, 171.00)	−0.826	.422
ALT (IU/L)	53.10 (22.5, 270.60)	15.50 (7.60, 139.10)	−1.579	.117
Total bilirubin (μmol/L)	34.20 (15.90, 79.80)	10.50 (5.21, 24.3)	−2.804	.004
Direct bilirubin (μmol/L)	13.6 (7.50, 34.20)	3.8 (2.52, 9.40)	−2.435	.014
Indirect bilirubin (μmol/L)	19.30 (9.20, 44.00)	5.80 (2.69, 14.90)	−2.922	.003
Urine volume (mL/h)	20.00 (7.50, 40.00)	0.00 (0.00, 5.00)	−2.606	.008
Duration of AKI at iniation of CRRT (day)	1.00 (1.00, 2.00)	3.00 (2.00, 7.50)	−1.330	.192
Fluid load (mL/day)	2871.00 (2040.00, 3390.00)	2170.50 (1803.25, 2685.00)	−1.682	.095
VIS score	21.00 (15.00, 27.20)	3.50 (0.75, 7.25)	−3.297	.001
Furosemide dose (mg/day)	200.00 (160.00, 240.00)	90.00 (80.00, 100.00)	−3.498	.000
Mean arterial pressure (mm Hg)	78.00 (72.15, 88.15)	86.60 (83.95, 93.65)	−2.569	.009
PH	7.47 (7.40, 7.52)	7.37 (7.29, 7.39)	−2.516	.010
CRRT duration (h)	59.00 (35.00, 86.00)	36.50 (18.75, 60.00)	−2.427	.014
CRRT dose (mL/kg.h)	28.17 (23.15, 33.34)	28.34 (20.55, 32.58)	−1.152	.256
Mortality (n)	7	2		.697

*Note*: Differences in continuous values between 2 groups were assessed with the Mann–Whitney *U* test. Differences in categorical factors were determined with the Fisher exact test. All of the tests were 2-sided, and differences were considered significant at *P* < .05.

AKI = acute kidney injury, ALT = alanine transaminase, BMI = body mass index, CRP = C-reactive protein, CRRT = continuous renal replacement therapy, PCT = procalcitonin.

There was no significant difference in age or sex between the 2 groups. The levels of direct and indirect bilirubin in patients with CSA-AKI before the application of CRRT were significantly greater than those in patients with AKI caused by other reasons. Compared with patients with AKI caused by other factors, patients with CSA-AKI were found to have lower mean arterial pressure, higher vasoactive-inotropic score, and greater daily diuretic dosages. However, the degree of kidney injury in patients with CSA-AKI was milder than that in patients with AKI caused by other reasons, characterized by lower serum creatinine levels and greater urine output. The levels of hemoglobin and pH before the application of CRRT were greater in patients with CSA-AKI than in patients with AKI caused by other reasons. Although there was no significant difference in the duration of AKI at initiation of CRRT between the 2 groups, there was a significant difference in the AKI stage at the time that CRRT started. Patients with AKI caused by other reasons received CRRT at AKI stage 3, while patients with CSA-AKI were prescribed CRRT earlier (4 patients received CRRT at AKI stage 1, and 6 patients were prescribed CRRT at AKI stage 2) (Tables 1 and 2).

**Table 2:** Comparison of AKI stage, anticoagulation methods, and CRRT mode between the 2 groups of patients at initiation of CRRT.

		Cardiac surgery-associated AKI (N = 28)	AKI without cardiac surgery (N = 12)	χ^2^	*P* value
AKI	AKI stage 1	4	0	5.793	.042
	AKI stage 2	7	0		
	AKI stage 3	17	12		
Anticoagulation	LMWH	14	0	20.755	.000
	Citrate	6	12		
	No anticoagulants	8	0		
CRRT mode	CVVHDF	16	4		.301
	CVVH	12	8		

*Note*: Differences in categorical factors were determined with the Fisher exact test. All of the tests were 2-sided, and differences were considered significant at *P* < .05.

AKI = acute kidney injury, CRRT = continuous renal replacement therapy, CVVH = continuous venovenous hemofiltration, CVVHDF = continuous venovenous hemodiafiltration, LMWH = low molecular weight heparin.

There was a significant difference in the use of CRRT for anticoagulation between patients with CSA-AKI and patients with AKI caused by other reasons. In this study, patients with AKI caused by other reasons were all treated with regional citrate anticoagulation (RCA). Six patients who underwent cardiac surgery were treated with RCA, and the other 22 patients were treated with low molecular weight heparin (LMWH) or without anticoagulants, of whom 10 patients were treated with oral warfarin at the same time (Table 2).

There was no significant difference in CRRT dose or CRRT mode (continuous venovenous hemofiltration vs continuous venovenous hemodiafiltration) between patients with CSA-AKI and patients with AKI caused by other reasons. However, the duration of CRRT in patients with CSA-AKI was longer than that in patients with AKI caused by other reasons.

### 3.2. Differences in performance between CSA-AKI patients who underwent RCA and those who did not

In this study, 6 patients with CSA-AKI were treated by CRRT with RCA, 14 patients were treated by CRRT with LMWH anticoagulation, and another 8 patients were treated by CRRT with no anticoagulation. There were no significant differences in alanine aminotransferase levels, bilirubin levels, platelets counts, free calcium levels, pH values, or sequential organ failure assessment scores before the application of CRRT between patients with and without RCA. Arterial blood samples were taken for gas analysis at 0 hours, 4 hours, and 8 hours after the start of CRRT. The results showed that there was no significant difference in the pH or lactate concentration between the patients with and without RCA. There were also no significant differences in intra-aortic balloon pump, cardiopulmonary bypass time, cardiac disease, the use of warfarin, or mortality between the patients with and without RCA. When comparing the side effects and effectiveness of the anticoagulant method between the patients with and without RCA, no significant differences were found in filter life or complications such as hemorrhagic complications or filter coagulation between the 2 groups (Tables 3 and 4).

**Table 3:** Comparison of clinical characteristics in patients with CSA-AKI who received CRRT with or without citrate anticoagulation.

Characteristics and measure	CSA-AKI without citrate anticoagulation (N = 22)	CSA-AKI with citrate anticoagulation (N = 6)	Z	*P* value
Cardiopulmonary bypass time (min)	121.00 (103.50, 176.5)	121.00 (110.00, 165.00)	0.549	.588
Age (years)	55.00 (48.50, 59.50)	63.00 (56.00, 64.50)	−0.869	.403
Free calcium (mmol/L)	1.15 (1.05, 1.42)	1.17 (1.10, 1.59)	−0.467	.659
PH	7.47 (7.40, 7.54)	7.45 (7.38, 7.49)	−0.029	.988
Platelets (×10^9^/L)	99.00 (63.00, 157.50)	93.00 (69.50, 146.50)	−0.028	.989
ALT (IU/L)	58.00 (33.45, 259.55)	45.00 (29.45, 2859.45)	−0.504	.643
Total bilirubin (μmol/L)	43.30 (25.80, 113.80)	28.40 (14.45, 83.20)	−0.896	.395
Direct bilirubin (μmol/L)	17.40 (10.95, 47.60)	9.30 (6.95, 26.40)	−1.120	.279
Indirect bilirubin (μmol/L)	23.10 (14.20, 47.95)	19.30 (7.40, 56.80)	−0.420	.692
SOFA	19.00 (17.00, 23.00)	19.00 (17.50, 20.50)	−0.450	.671
Filtration fraction at 0 h	24.64 (21.87, 29.50)	29.20 (22.58, 34.08)	−0.392	.723
Lactate at 0 h (mmol/L)	3.20 (2.00, 4.20)	1.60 (1.05, 3.15)	−1.682	.096
PH at 0 h	7.46 (7.39, 7.53)	7.39 (7.35, 7.42)	−0.787	.450
Lactate at 4 h (mmol/L)	2.80 (1.90, 5.80)	1.40 (1.20, 3.05)	−1.569	.122
PH at 4 h	7.43 (7.37, 7.48)	7.41 (7.31, 7.43)	−0.309	.774
Lactate at 8 h (mmol/L)	3.00 (1.85, 4.90)	1.00 (0.95, 2.40)	−1.653	.102
PH at 8 h	7.43 (7.38, 7.48)	7.40 (7.35, 7.42)	−1.038	.315
Filter life (h)	24.00 (12.00, 24.00)	24.00 (11.00, 39.00)	−0.265	.807
IABP	11	1		.196
Warfarin	7	3		.634
Hemorrhagic complication	5	0		.553
Filter coagulation	8	3		.653
Mortality (n)	5	2		.622

*Note*: Differences in continuous values between 2 groups were assessed with the Mann–Whitney *U* test. Differences in categorical factors were determined with the Fisher exact test. All of the tests were 2-sided, and differences were considered significant at *P* < .05.

ALT = alanine transaminase, BMI = body mass index, CRP = C-reactive protein, CRRT = continuous renal replacement therapy, CSA-AKI = cardiac surgery-associated acute kidney injury, IABP = intra-aortic balloon pump, PCT = procalcitonin, SOFA = sequential organ failure assessment.

**Table 4:** Comparison of cardiac disease in patients with CSA-AKI who received CRRT with or without citrate anticoagulation.

	Patients without citrate anticoagulation (N = 22)	Patients with citrate anticoagulation (N = 6)	χ^2^	*P* value
Valvular heart disease (n)	13	4	2.396	.823
Aortic dissection (n)	3	2		
Coronary atherosclerotic heart disease (n)	1	0		
Infective endocarditis (n)	2	0		
Congenital heart disease (n)	3	0		

*Note*: Differences in categorical factors were determined with the Fisher exact test. All of the tests were 2-sided, and differences were considered significant at *P* < .05.

CRRT = continuous renal replacement therapy, CSA-AKI = cardiac surgery-associated acute kidney injury.

## 4. Discussion

Despite the clear definition of AKI, the timing of initiation of CRRT remains at the discretion of the nephrologist specialist based on the clinical situation. This study revealed that patients with CSA-AKI received CRRT at an earlier AKI stage than patients with AKI caused by other reasons. This may be due to the consideration of relative fluid overload after cardiac surgery. Cardiac surgery is often combined with low cardiac output and impaired postoperative ventricular diastolic function.^[6–10]^ In this study, patients with CSA-AKI were found to have higher vasoactive-inotropic score and greater diuretic dosages, indicating the presence of cardiac dysfunction and low diuretic responsiveness. Some studies have shown that perioperative fluid overload during cardiac surgery is associated with a poor prognosis and is a major risk factor for multiple organ failure, including AKI.^[11,12]^ On the other hand, following cardiac surgery, patients are prone to hemodynamic instability, hypotension, and increased risk of liver damage caused by hepatic congestion and ischemia reperfusion. Therefore, CRRT is usually performed at AKI stage 2 or earlier to provide rapid fluid control.^[13–15]^ Patients with AKI caused by other reasons were all in stage 3 at the time of initiation of CRRT. The degree of anemia and acidosis was more serious. This may be because the patients had no heart disease requiring surgery, cardiac function was relatively good, and blood pressure was relatively stable at the early stage of AKI. Conservative drug treatment is currently preferred in clinical practice. CRRT was needed when the patient’s condition was aggravated, especially complicated by serious infection or acidosis, and at this time, the patient was in a relatively late stage of AKI.

We found that there was a significant difference in the use of CRRT for anticoagulation between patients with CSA-AKI and patients with AKI caused by other reasons. Good anticoagulation is the key factor to ensure the implementation of CRRT. At present, the anticoagulation methods frequently used in CRRT include RCA, LMWH anticoagulation, and no anticoagulant regiments. At the 2012 Kidney Disease Improving Global Outcomes Conference, RCA was recommended as the preferred anticoagulant for patients without contraindications.^[7]^ In this study, patients with CSA-AKI were more likely to use LMWH as an anticoagulant. This may be due to the risk of citrate accumulation in some conditions, such as severe circulatory dysfunction caused by cardiac function failure after surgery and severe hepatic injury caused by hepatic congestion or ischemia–reperfusion. In addition, there is a risk of hypocalcemia. Hypocalcemia in patients after cardiac surgery can likely lead to severe cardiovascular inhibition and congestive heart failure with a reduced effect of positive inotropic drugs.^[16,17]^ Therefore, LMWH is a relatively safe anticoagulant for patients without obvious bleeding risk. However, RCA is preferred in the absence of contraindications in patients with AKI caused by other reasons. The conventional dose of warfarin can be used to prevent thrombosis under the RCA, while the dose of warfarin under LMWH should be reduced appropriately.^[18,19]^

The anticoagulation effect of CRRT in patients with CSA-AKI was further analyzed. Only 6 patients used RCA. In this study, the clinical characteristics of patients and the side effects and effectiveness of anticoagulant methods were compared between patients with and without RCA. We did not find any differences in liver function, platelet count, or cardiopulmonary bypass time between the 2 groups of patients before initiating CRRT. No significant differences were found between the 2 groups in terms of filter life or the occurrence of coagulation or bleeding complications. Since this was only a single-center retrospective study with a small sample size, it was still unclear whether the 2 anticoagulation methods were equivalent in terms of side effects and effectiveness. In this study, some of the patients who chose either the RCA or non-RCA regimens received concurrent warfarin. It has been reported that warfarin can be used to prevent thrombosis after cardiac surgery, but it is recommended to reduce the dose of warfarin when it is used together with LMWH.^[18,19]^

## 5. Limitations

The current study had several limitations. Firstly, this study was a retrospective single-center study with a limited sample size. Further prospective observations in larger sample sizes from multiple centers are needed. Additionally, the lack of echocardiography during AKI occurrence after cardiac surgery and CRRT intervention made it challenging to accurately assess cardiac function. Furthermore, the CSA-AKI patients underwent different types of cardiac surgeries, including heart valve surgery, coronary artery bypass grafting surgery, and congenital heart disease surgery, which may have influenced the outcome indicators. Future studies with larger samples should consider conducting further analysis.

## 6. Conclusions

Due to the presence of cardiac dysfunction and low diuretic responsiveness, CRRT is initiated earlier in patients with CSA-AKI than in patients with AKI caused by other reasons. Although RCA is recommended as the preferred anticoagulant for patients without contraindications, patients with CSA-AKI often have circulatory dysfunction and severe liver damage, so the risk of citrate accumulation is greater. In addition, hypocalcemia caused by citrate can also increase the burden on the heart, so whether RCA should be used should be determined according to the condition of the individual patient.

## Acknowledgments

It is grateful for all the nurses of the CRRT team for their therapeutic help.

## Author contributions

**Conceptualization:** Xianglian Li.

**Writing – original draft:** Jian Li, Yi Li, Liwen Mo, Fan Zhang, Yue Cheng.

**Writing – review & editing:** Yi Li, Liwen Mo, Fan Zhang, Yue Cheng, Tao Wang.
